# Morphological dissection and cellular and transcriptome characterizations of bamboo pith cavity formation reveal a pivotal role of genes related to programmed cell death

**DOI:** 10.1111/pbi.13033

**Published:** 2018-12-09

**Authors:** Lin Guo, Xuepeng Sun, Zhongru Li, Yujun Wang, Zhangjun Fei, Chen Jiao, Jianyuan Feng, Dingfan Cui, Xingyu Feng, Yulong Ding, Chunxia Zhang, Qiang Wei

**Affiliations:** ^1^ Co‐Innovation Center for Sustainable Forestry in Southern China Nanjing Forestry University Nanjing Jiangsu China; ^2^ Boyce Thompson Institute for Plant Research Cornell University Ithaca NY USA; ^3^ International Education College Nanjing Forestry University Nanjing Jiangsu China; ^4^ Bamboo Research Institute Nanjing Forestry University Nanjing Jiangsu China

**Keywords:** pith cavity, programmed cell death, fast growth, reactive oxygen species, calcium, asymmetrical growth

## Abstract

Pith cavity formation is critical for bamboo to overcome the bending force during its fast growth; however, the underlying molecular mechanisms remain largely unknown. Multiple approaches, including anatomical dissection, mathematical modelling and transcriptome profiling, were employed in this study to investigate the biology of pith cavity formation in bamboo *Pseudosasa japonica*. We found that the corruption of pith tissue occurred sequentially and asymmetrically from the top‐centre of the internode down to the bottom, which might be caused by the combined effects of asymmetrical radial and axial tensile forces during shoot‐wall cell elongation and spiral growth of bamboo internodes. Programmed cell death (PCD) in pitch manifested by TUNEL positive nuclei, DNA cleavage and degraded organelles, and potentially regulated by ethylene and calcium signalling pathway, ROS burst, cell wall modification, proteolysis and nutrient recycle genes, might be responsible for pith tissue corruption of *Ps. japonica*. Although similar physiological changes and transcriptome profiles were found in different bamboo species, different formation rates of pith cavity were observed, which might be caused by different pith cells across the internode that were negatively correlated with the culm diameter. These findings provided a systematical view on the formation of bamboo pith cavity and revealed that PCD plays an important role in the bamboo pith cavity formation.

## Introduction

Bamboo is an important forest resource in the world. It has been used for afforestation, papermaking, construction, food and environment protection (Wei *et al*., 2015). As a green, renewable and fast growth plant, bamboo has attracted much attention worldwide.

Although bamboo has been used for many purposes, little is known about its fundamental biological processes (Wei *et al*., 2017). For a long time, the molecular research of bamboo mainly follows the studies of model plants (Wei *et al*., 2013). Recently, with the advancement of next generation sequencing technologies, several studies have reported to explore bamboo transcriptome profiles in several important biological processes such as fast growth, flowering and primary thickening growth (Gao *et al*., 2014; He *et al*., 2013; Li *et al*., 2018; Liu *et al*., 2012; Peng *et al*., 2013; Shih *et al*., 2014; Wei *et al*., 2017, 2018; Zhang *et al*., 2012). Meanwhile, the genetic transformation system of bamboo has been successfully established (Qiao *et al*., 2014; Ye *et al*., 2017), and several groups have made progress in increasing the callus regeneration efficiency, which is important for establishing an efficient transgenic system of bamboo (Wei *et al*., 2015). These works have opened a new window for the molecular research of bamboo developmental processes.

Unlike the uniform internode shape of other monocotyledon grass species, bamboo internodes display high morphological diversity. For example, the diameter of bamboo culm ranges from about 0.3 cm to over 30 cm (Wei *et al*., 2017). Our previous work revealed that pith, which has been long ignored by bamboo researchers since it will eventually die to form a cavity and therefore has not been considered valuable, plays an unexpected important role in promoting the primary thickening growth and driving the culm size evolution of bamboo (Wei *et al*., 2017). A few candidate genes involved in the regulation of the pith initiation were identified (Wei *et al*., 2017). However, so far little attention has been paid to the postinitiation development of pith, especially the formation of pith cavity, which occurs at the final developmental stage and is important for establishing the hollow culm to efficiently conquer the bending force during the fast growth of the bamboo stem (Thompson, 1945). So far, there is only a very preliminary view in the bamboo research field, that the formation of pith cavity of bamboo culm is resulted from different growth rates between the shoot wall cells and the pith cells during the fast growth of bamboo shoots, similar to the schizogenous aerenchyma formation in some plants such as intercellular gas space formation in the leaf petiole of *Sagittaria trifolia* (Liang *et al*., 2008; Ni *et al*., 2014).

To investigate molecular mechanisms underlying the formation of bamboo pith cavity, we investigated the cellular and physiological changes as well as the corresponding transcriptome profiles during pith tissue collapse of *Pseudosasa japonica*, a bamboo species with slow pith tissue collapse process, and convenient for collecting pith tissues at different developmental stages. We then investigated the cellular changes and molecular basis of the pith cavity formation in several other bamboo species. We found that different bamboo species shared similar cellular and molecular mechanisms underlying the pith cavity formation. However, these bamboo species displayed morphological variations of pith cavity formation. The possible underlying cellular basis was also explored in this study.

## Results

### Morphological and anatomical characterization of bamboo pith cavity development

**Figure 1:**
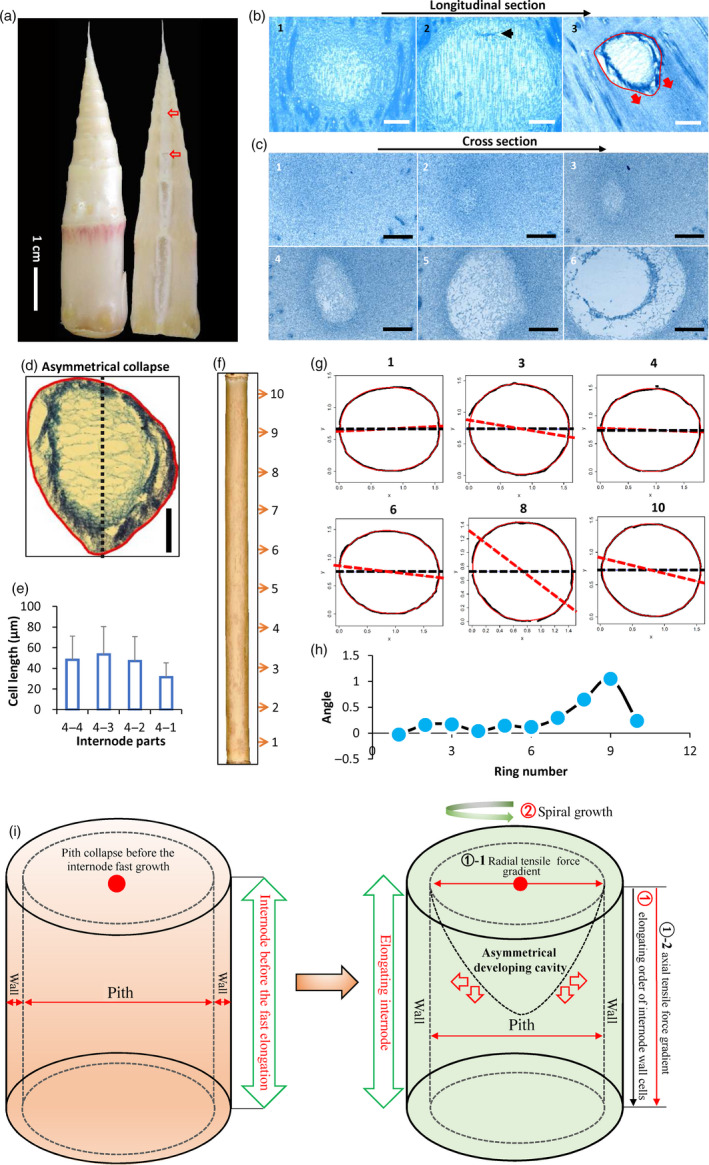
Morphological analysis of pith cavity formation in bamboo. (a) Longitudinal sections of a bamboo shoot of *Ps. japonica*. Red arrows indicate broken pith tissues. (b, c) Continuous longitudinal (b) and cross sections (c) from top to bottom of the *Ps. japonica* shoot shown in (a). Closed red line indicates the pith cavity from the axial view, and red arrows indicate the broken trend of pith tissue. Scale bars: 100 μm. (d) An extracted asymmetrically formed pith cavity of *Ps. japonica*. Scale bars: 100 μm. (e) Parenchymal cell lengths along the second internode of *Ps. japonica* with a length of 4‐cm form bottom to top part. (f) A mature second internode of *Ps. japonica* culm. The numbers indicate the parts used for the mathematical analysis. (g) Merged pictures of the outer contours (black solid lines) and the predicted rings (red solid lines). Numbers at the top indicate the corresponding parts in (f). The black dashed line represents the direction of the *x*‐axis and the red dashed line represents the direction of the major axis. (h) Angles between the major axis and *x*‐axis of the 10 rings indicated in (f). (i) A hypothetical model of bamboo pith cavity formation. The gradients of radial and axial tensile forces caused by the sequential elongation of shoot wall cells from top to bottom first result in the easier separation of broken pith tissue in the top‐centre part of the internode, and the spiral growth of the internode further causes the asymmetrical development of the pith cavity formation during the subsequent internode elongation process.

Morphological analysis revealed that pith cavity started to form before the fast growth of internode and continued to form during the fast growth in *Ps. japonica* (Figure 1a). Cytological analysis showed that pith tissue in *Ps. japonica* was first corrupted in the central zone on the top of the internode before fast elongation, and pith cavity was progressively formed during the elongation of internode (Figure 1b). During the pith cavity formation process, pith cells gradually became enlarged and irregular, and finally disrupted (Figure 1c). In addition, we also found that pith cavity formation of bamboo was asymmetrical (Figure 1d).

To investigate possible factors that resulted in the pith cavity formation of bamboo plant after pith collapse in the top‐center part, we measured the cell lengths along an internode with a length of 4 cm, and found that cells in the top two‐centimetre part were slightly longer than those in the bottom two‐centimetre part (Figure 1e), indicating that cells in the top part of the internode elongated earlier than those of cells in the bottom part.

To investigate factors causing the asymmetrical pith cavity formation, we then used a mathematical method to describe the internode elongation pattern of *Ps. japonica* (Figure 1f). As shown in Figure 1g, the superellipse equation (Gielis, 2003; Shi *et al*., 2015) could precisely describe the outlines of the cross sections in different parts of the internode of *Ps. japonica*. As expected, we found that the angles between the major axis and the horizontal axis of the fitted ellipses progressively increased from first to third rings, and subsequently dropped in the fourth ring, and then dramatically increased from the fourth to the ninth rings, which displayed a spiral growth pattern (Figure 1h).

On the basis of the above morphological and anatomical results, we proposed a model of pith cavity formation in bamboo. We thought that pith cells first broke in the top and centre zone of the pith tissue adjacent to node before the fast growth of the internode, and then the corruptions spread into the peripheral and bottom pith tissues prior to and during the internode elongation (Figure 1i). This pith postcorruption pattern might be caused by the axial and radial as well as the asymmetrical tensile force gradients that were triggered by special cell elongation pattern of the bamboo internode, that is, cells elongated sequentially from top to bottom parts, and the internode displayed a spiral growth pattern (Figure 1i).

### Cellular characterization of pith cell death during the formation of pith cavity of *Ps. japonica*

**Figure 2:**
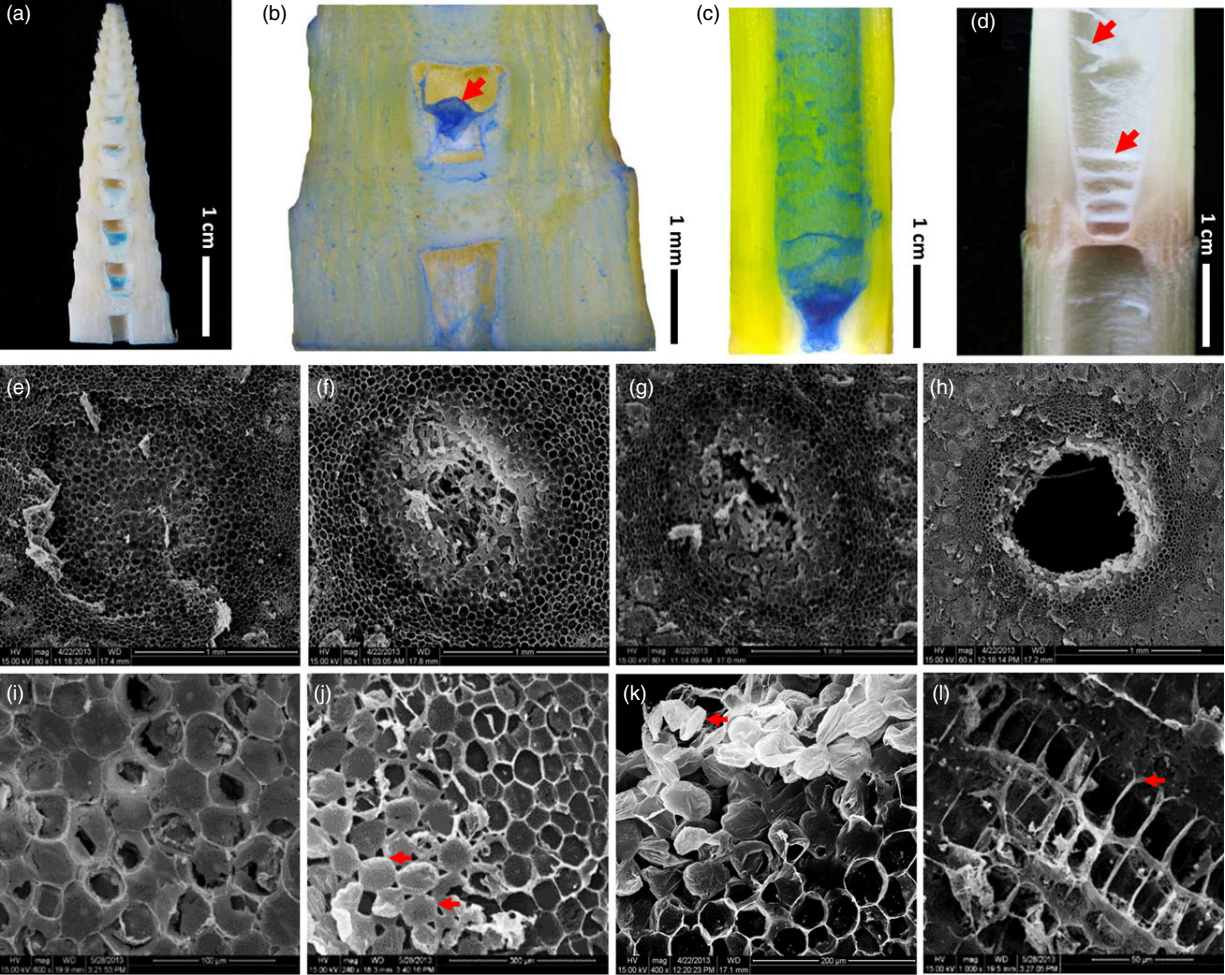
Progressive death of pith cells results in the pith cavity of *Ps. japonica*. (a) Trypan blue staining of pith tissues in a bamboo shoot with a height of ~4.5 cm and without a bamboo sheath. (b) A close look of trypan blue stained pith tissue. (c) Trypan blue stained pith cells in a nearly elongated internode. (d) Dead pith cells finally became dry and membrane like tissues. (e–h) Morphological observations of pith cavity formation by scanning electron microscope discovered that pith cavity was formed from the central zone (e) and progressively expanded during the internode elongation (f–h). Four morphological changes, enlarging (i), separation (j), shrinking (k) and collapse (l), of pith cells during their dying process were observed. Red arrows in (j), (k) and (l) indicate extended intercellular spaces, shrinking pith cells and pith cell corpses, respectively.

Trypan blue staining which can specially label dead cells clearly showed that pith cells died during the formation of pith cavity (Figure 2a–c), and turned into dry membrane structures eventually (Figure 2d). Scanning electronic microscope (SEM) observations of pith cell morphology during the pith cavity formation of *Ps. japonica* obtained similar results to what light microscope discovered, that pith cavity was formed from the central zone and progressively expanded during the internode elongation (Figure 2e–h). Pith cells would then go through enlarging (Figure 2i), separation (Figure 2j), shrinking (Figure 2k) and crush (Figure 2l) stages during the entire dying process.

**Figure 3:**
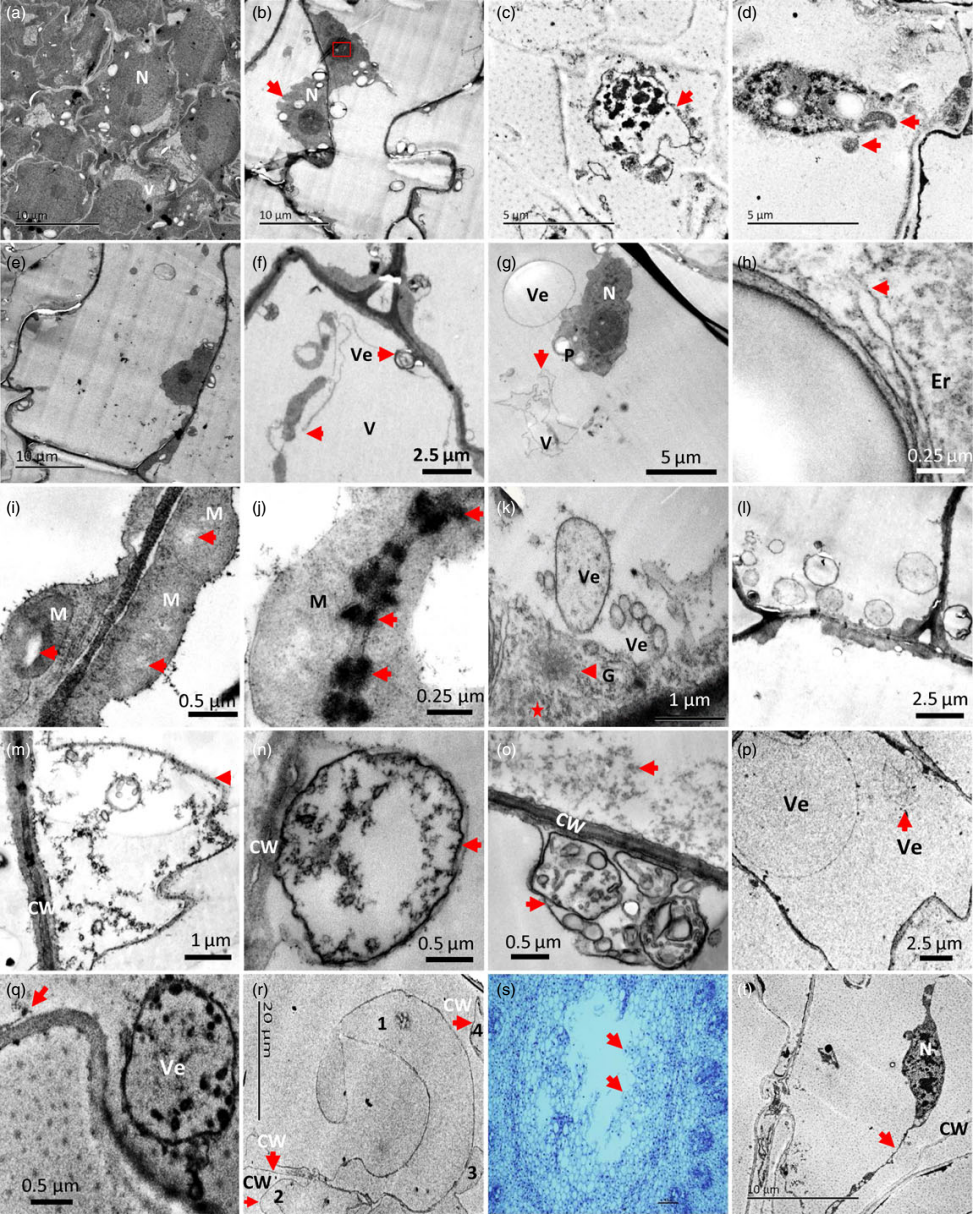
Ultrastructure of nucleus and cytoplasmic changes in dying pith cells of *Ps. japonica*. (a) Normal round nucleuses in the pith cells with the division ability. (b) Lobbing of nucleus (red arrow) began to appear in the pith cell at the early stage of pith cavity formation. Red rectangle indicates the vacuolization of nucleolus. (c) Chromatin condensing nucleus with intact nuclear envelope (pointed by red arrow) in the dying pith cells. (d) Dismantled nucleus with apoptosis body like structures (red arrows). (e) A large centre vacuole was formed in the mature pith cell. (f) Tonoplast ruptured (red arrow) and vesicles present near the plasma membrane. (g) Collapse of vacuole (red arrow). (h) Endoplasmic reticulum became broken (red arrow). (i) Vacuolization (red arrow) emerged in the centre of mitochondrion. (j) Plasmodesmata became indistinct (red arrow). (k) Golgi apparatus became unclear and began to degrade, and cytoplasm degraded apparently (star). (l) An apparent increase of vesicles was found in the pith cells. (m, n) Cell wall became thin and collapsed, which were degraded into granule materials surrounded by the secondary vacuoles formed from the cell membrane. (o) Various materials such as vesicles, membrane structures, tubulars and granules were found in the secondary vacuoles. (p) Secondary vacuoles and their contents appeared to degrade last. (q) Plasma membrane began to rupture (red arrow). (r) Degraded and broken cell walls in the inner surface of the pith cavity (red arrows). Numbers indicate different cells. (s) A forming pith cavity from the cross‐section view. Red arrows indicate cells for TEM observation. (t) Plasmolysis found in some dying cells around the pith cavity. CW, cell wall; Er, endoplasmic reticulum; G, Golgi apparatus; M, mitochondria; N, nucleus; V, vacuole; Ve, vesicle.

Transmission electronic microscope (TEM) was used to obtain more information about the pith cell death from the perspective of the subcellular structure. The observations revealed that during the cell dying process, the earliest morphological sign of nuclear degradation was reflected by the appearance of vacuolated nucleolus and crenulated nuclear membrane (Figure 3a,b). This was followed by chromatin condensing with intact nuclear membrane (Figure 3c). The nucleus was immediately dismantled after this step (Figure 3d). Furthermore, TEM observations revealed that with the differentiation of cells, small vacuoles were combined to form larger central vacuoles (Figure 3a,e). The first indication of programmed cell death (PCD) in pith cells was the ruptured tonoplast (Figure 3f,g). Subsequently, the organelles began to degrade, as indicated by the broken endoplasmic reticulum (Figure 3h) and the vacuolization of mitochondria (Figure 3i). The plasmodesmata (Figure 3j) and golgi apparatus (Figure 3k) became disrupted and indistinguishable. Degraded cytoplasmic components were found in the cell lumens (Figure 3k). Following this step, an increased number of vesicles aggregated in the cytoplasm (Figure 3l,m), and later, secondary vacuoles formed by invaginations of the plasma membrane, which contained granule materials possibly derived from the cell wall, began to emerge as the cell wall became thin and disrupted (Figure 3n). Vesicles, membrane structures, tubulars and granules were also found in the secondary vacuoles (Figure 3o), which appeared to be degraded eventually (Figure 3p). Later, ruptured plasma membrane was observed (Figure 3q). The disruption of the cell wall was the last structural event of PCD during the pith cell death process. Thin, broken and separated cell walls were clearly present in the inner surface of the pith cavity (Figure 3r). In addition, plasmolysis was detected in some cells around the forming pith cavity (Figure 3s,t).

### DNA cleavage, RNA degradation and TUNEL positive nuclei formed during the pith cell death of *Ps. japonica*

**Figure 4:**
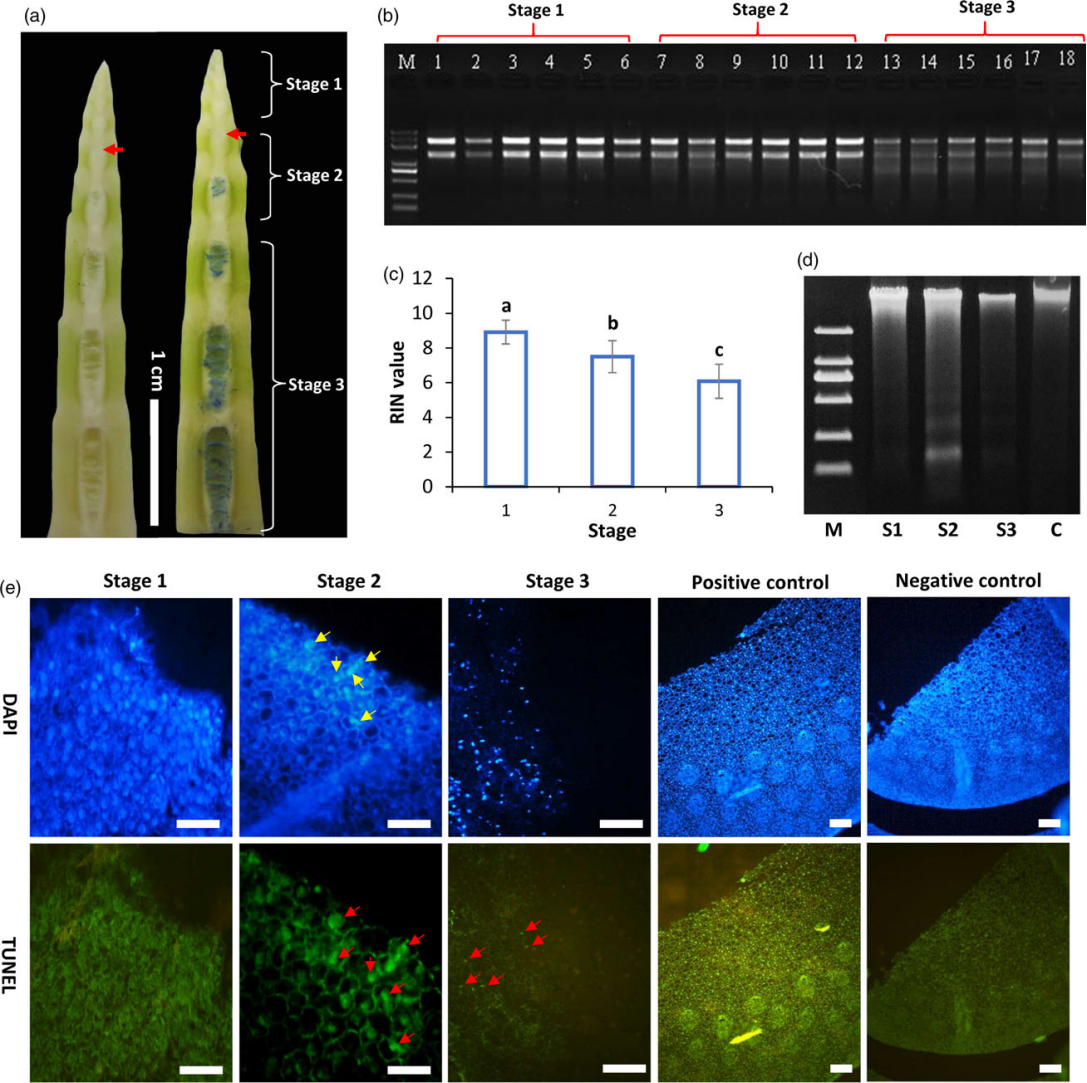
DNA cleavage and TUNEL assay of pith cells in *Ps. japonica*. (a) Pith tissues at three stages were collected for RNA and DNA ladder detection. Red arrows indicate the broken pith tissue. (b) RNA degradation of pith cells at different developmental stages described in (a). (c) RIN values of pith cells at three different stages. (d) DNA degradation of pith cells at different developmental stages described in (a) using agarose gel electrophoresis. DNA from leaf indicated by letter C was used as the control. S1, stage 1; S2, stage 2; S3, stage 3. (e) Positive TUNEL nuclei (red arrows) were found during pith cell dying process. Scale bars: 100 μm. Negative controls were sections incubated without adding the terminal transferase, and positive controls were those incubated with DNase I. Yellow arrows indicate the DAPI stained condense nuclei.

The above cytological observations suggested that the pith cavity formation is highly possibly a process of programmed cell death. To further test this hypothesis, DNA and RNA were isolated from the pith tissues at three different stages (Figure 4a) and separated by agarose gel electrophoresis. RNAs from the stage 2 (S2) pith tissues that began to break had a little smeared ladder but were degraded obviously in the stage 3 (S3) at which pith cavity actively formed, while RNAs from stage 1 (S1) unbroken tissues had clear and sharp bands with a high RNA integrity number (RIN) value (Figure 4b,c). RIN values progressively decreased from S1 to S3 (Figure 4c), which were correlated well with the electrophoresis result. DNA ladder was not detected for DNA isolated from the leaf (non‐PCD tissue) or pith at stage 1 (Figure 4d). Smeared DNA ladders were detected in pith tissues at stage 2 and 3 (Figure 4d).

Terminal deoxynucleotidyl transferase dUTP nick end labeling (TUNEL) assay and 4′, 6‐diamidino‐2‐phenylindole (DAPI) staining were further performed to investigate the DNA cleavage. In the S1 pith cells, DNA uniformly spread in the nuclei and no TUNEL‐positive nuclei were detected, but clear TUNEL‐positive nuclei with DNA condensing (indicated by bright DAPI stained nuclei) started to appear in S2 pith cells around the initial pith cavity, while faint TUNEL‐positive nuclei were detected in the S3 pith cells (Figure 4e). For sections that were treated with DNase I (positive control), almost all the nuclei were TUNEL‐positive, and no positive labelling was observed in the negative controls which are sections incubated without terminal transferase enzyme (Figure 4e).

### Transcriptome profiling analysis of pith cavity formation in *Ps. japonica*

To explore the molecular mechanisms underlying the pith cavity formation of bamboo shoots, we performed transcriptome sequencing of pith tissues at aforementioned three stages of *Ps. japonica*. A total of approximately 304 million raw read pairs were generated. After removing adaptor and low‐quality sequences, we obtained a total of ~275 million high quality read pairs with a total of ~82 Gb sequences (Table S1). These reads were *de novo* assembled into 157 850 unigenes with an N50 length of 1981 bp. The completeness of the assembled unigenes were then checked using BUSCO (Simao *et al*., 2015; Waterhouse *et al*., 2017), and the result indicated that 90.1% of the core conserved plant genes were captured by the assembled unigenes, and 75.6% were completely captured (Table S2).

Transcriptome profiles of most samples had high correlations (Pearson's *r* > 0.8) within biological replicates, suggesting the high quality of the RNA‐Seq data (Table S3). Comparison of transcriptome profiles between the pith tissues of *Ps. japonica* at the three developmental stages (Figure 4a) identified a large number of differentially expressed genes (DEGs). A total of 13 199 DEGs were identified between S2 and S1 pith tissues, of which 6277 were down‐regulated and 6,922 were up‐regulated in the pith tissues at the S2 stage (Table S4). Only 1163 DEGs (936 up‐regulated and 237 down‐regulated in S3) were found between S3 and S2 pith cells. We also compared the transcriptome profiles between S3 and S1 pith tissues, and 23 259 DEGs were discovered, of which 12 440 were up‐regulated and 10 859 were down‐regulated in S3 (Table S4).

**Figure 5:**
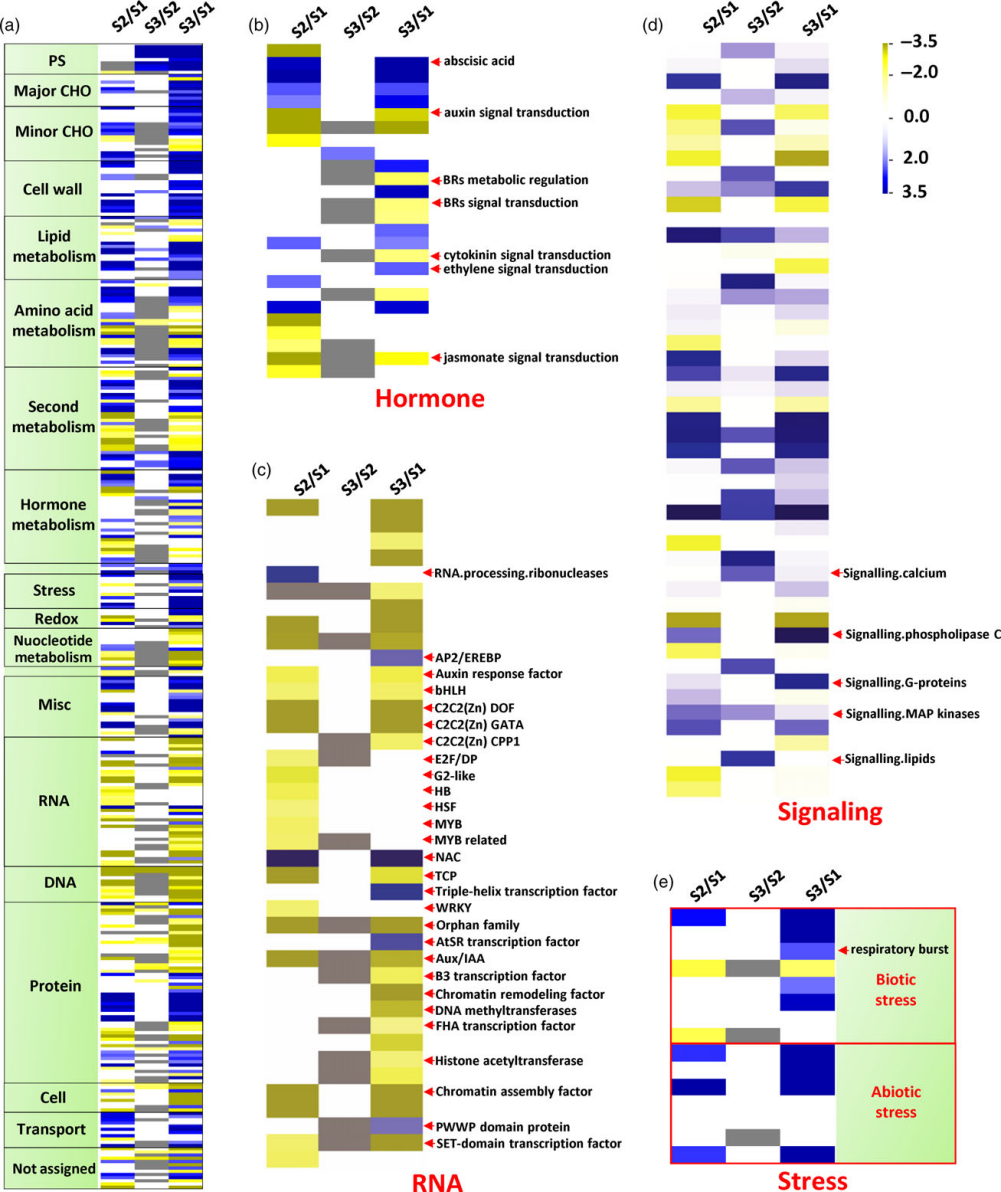
Ethylene, Ca^2+^ signalling and ROS burst related genes were up‐regulated in dying pith cells of *Ps. japonica*. (a) Overview of differentially expressed genes in pith cells during the formation of pith cavity. Hormone (b), RNA (c), signalling (d) and stress (e) related differentially expressed genes are shown. The figure was generated using PageMan of the MapMan program. S2/S1, S3/S2 and S3/S1 indicate ratios of gene abundance in the pith tissue between the two different stages.

MapMan (Thimm *et al*., 2004) analyses revealed that S3 and S2 pith cells had similar transcriptome profiles, while dramatical changes in various cellular processes were found between S3/S2 and S1 pith cells (Figure 5a). For example, genes related to auxin, brassinosteroid, cytokinin and jasmonate signal transduction were down‐regulated in the S3/S2 pith cells, while ethylene and abscisic acid pathways were significantly up‐regulated in the S3/S2 pith cells (Figure 5b). Most of DEGs related to RNA were also significantly down‐regulated in the S3/S2 pith tissues except those encoding AP2/EREBP, NAC, triple‐helix and AtSR transcription factors (Figure 5c). In the ‘Signaling’ category, DEGs related to cadmium, phospholipase C, G‐proteins and MAP kinase signalling pathways were significantly enhanced in the S3/S2 pith cells (Figure 5d). In addition, respiratory burst related genes were also up‐regulated in the S3/S2 cells (Figure 5e), indicating that reactive oxygen species (ROS) might participate in the pith cavity formation process.

**Figure 6:**
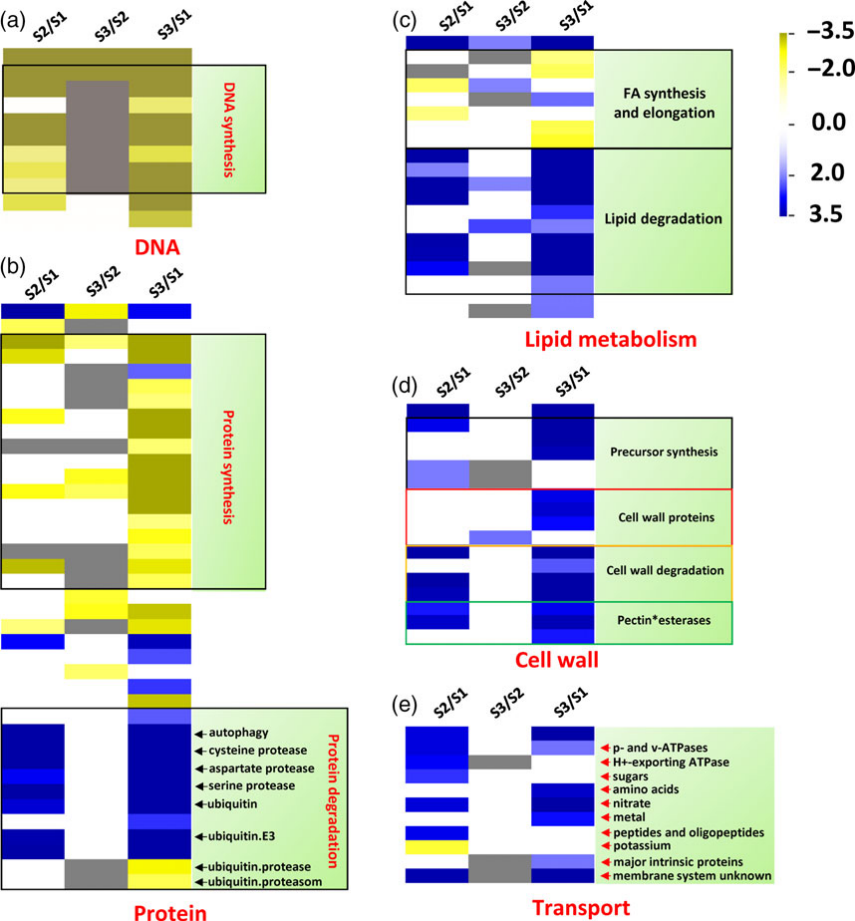
Macromolecule degradation and transport‐related genes were up‐regulated, while biosynthesis‐related genes were down‐regulated during the development of pith cavity of *Ps. japonica*. (a) Overview of differentially expressed genes related to DNA (a), protein (b), lipid metabolism (c), cell wall (d) and transport (e). The figure was generated using PageMan of the MapMan software.

We also investigated the downstream functional genes differentially expressed between S3/S2 and S1 pith tissues, and found that genes involved in DNA synthesis and repair, protein, lipid synthesis were dramatically down‐regulated in the S3/S2 pith cells in contrast to the up‐regulation of genes in their degradation pathways (Figure 6a–c). Interestingly, cell wall modification and degradation related genes were both up‐regulated in the S3/S2 pith cells (Figure 6d). In addition, most of transport related DEGs were up‐regulated except potassium transporter genes (Figure 6e).

**Figure 7:**
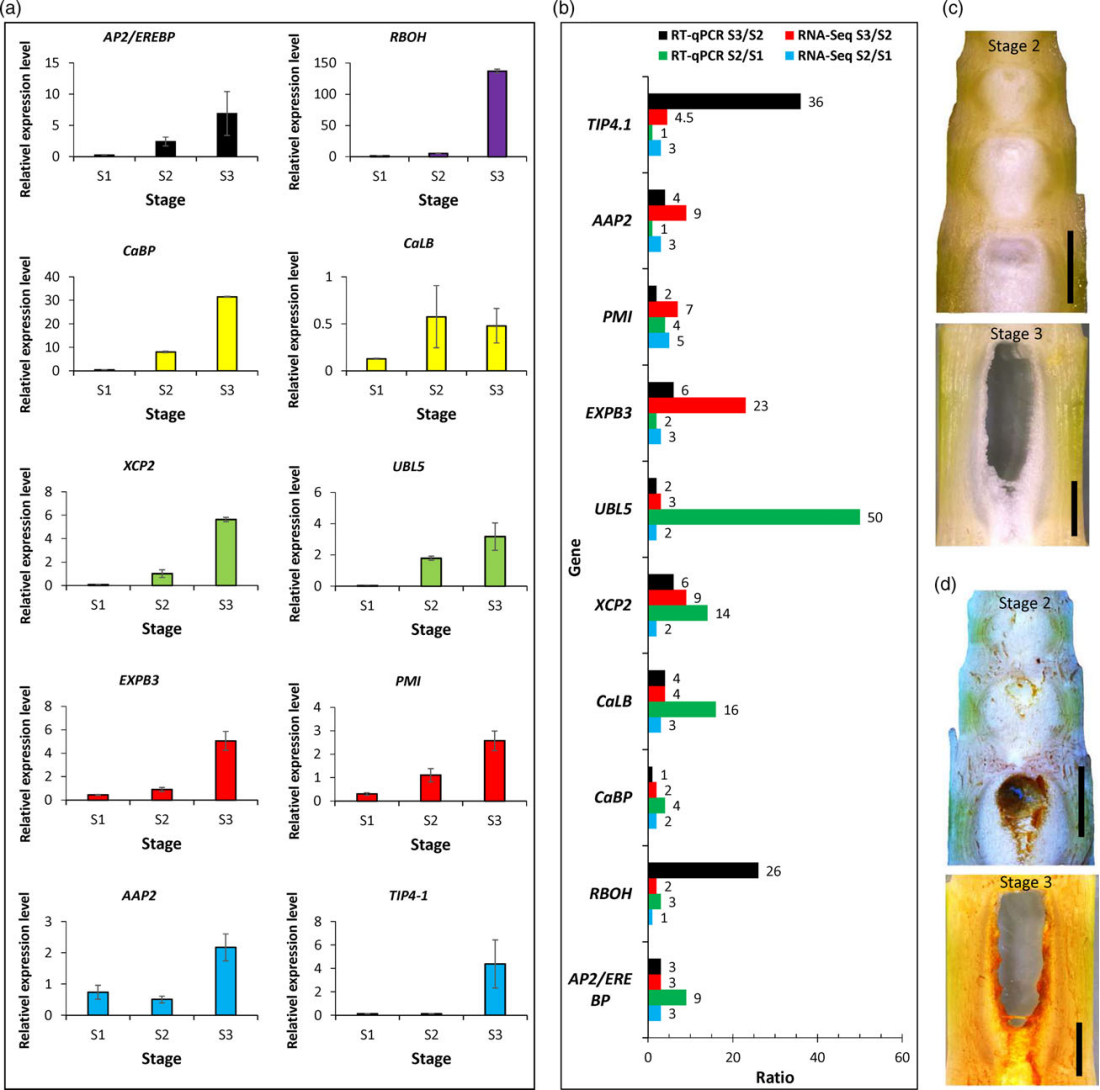
qRT‐PCR analysis of ten candidate genes during the pith cavity formation of *Ps. japonica*. (a) Relative expression levels of ten candidate genes in the pith cells during pith cavity formation. Data are means ± SD. (b) Ratios of gene expression levels analysed by RNA‐Seq and qPCR. Numbers at the right of the histogram indicate the average ratio values. *AP2/EREBP*: ethylene response factor; *RBOH*: respiratory burst oxidase protein; *CaBP*: calcium‐binding EF‐hand family protein; *CaLB*: calcium‐dependent lipid‐binding protein; *XCP2*: xylem cysteine peptidase 2; *UBL5*: ubiquitin‐like protein 5; *EXPB3*: Beta expansin; *PMI*: pectin methylesterase inhibitor; *AAP2*: amino acid permease 2; *TIP4‐1*: tonoplast intrinsic protein 4‐1. (c) Vertical‐sections of *Ps. japonica* pith tissues at stage 2 and stage 3 that were treated with ddH_2_O, and stained brown with DAB to visualize H_2_O_2_ (d).

The transcriptome data clearly revealed that ethylene, calcium, ROS and cell wall modification as well as nutrition recycle genes might constitute a molecular network regulating the pith cell death of *Ps. japonica*. To further validate our RNA‐Seq expression profile data, we performed quantitative real time PCR (qPCR) assays on ten randomly selected candidate genes, including one in the ethylene signalling pathway, one respiratory burst gene, two in the calcium signalling pathway, two in protein degradation, two in nutrition transport and two in cell wall modification. The results showed that all of those genes were dramatically up‐regulated in the stage 2 or stage 3 pith cells (Figure 7a), and although the exact fold changes of the selected unique transcripts varied between RNA‐Seq expression and qPCR analyses, the trend of gene expression change was largely similar (Figure 7b). Furthermore, to confirm the involvement of ROS in pith cell death, we determined the presence of ROS species H_2_O_2_ in prepith‐cavity cells. As expected, H_2_O_2_ was abundant in the precavity pith cells of *Ps. japonica* (Figure 7c,d).

### Pith cavity formation in other bamboo species

**Figure 8:**
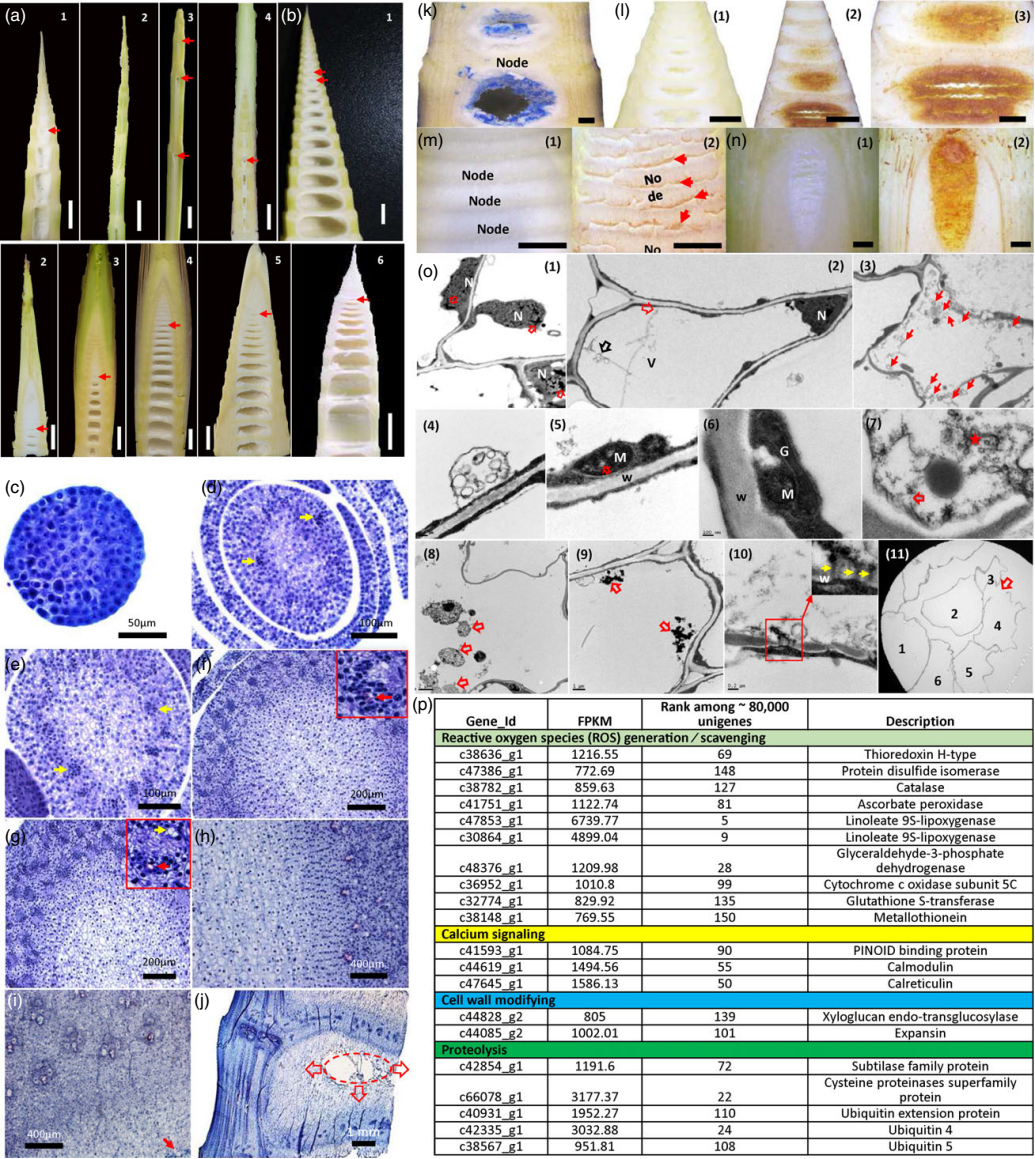
Morphological, physiological and molecular characterization of pith cavity formation in different bamboo species. (a) Longitudinal sections of bamboo shoots of *Pseudosasa japonica* (1), *Semiarundinaria densiflora* (2), *Indocalamus longiauritus* (3) and *Chimonobambusa sichuanensis* (4). Red arrows indicate the broken pith tissues. (b) Longitudinal sections of bamboo shoots of Moso bamboo (1), *Ph. propinqua* (2), *Ph. arcana* ‘Luteosulcata’ (3), *Ph. iridescens* (4), *Ph. bissetii* (5) and *Ps. polymorphum* (6). Red arrows indicate the broken pith tissues. (c–j) Pith development of Moso bamboo shoot. (c) Apical meristem of Moso bamboo shoot. (d) Pith cells in Moso bamboo appeared apparently when rib meristems first appeared (pointed by yellow arrows). (e) Differentiation of pith cells when the innermost rib meristems appeared apparently (pointed by yellow arrows). (f) Differentiation of pith cells when the rib meristems first differentiated into the protoxylem vessels (pointed by red arrow). Red rectangle, a close look of a developing vascular bundle. (g) Pith tissue cells in the centre displayed a strong vacuolization phenotype when the rib meristems were first differentiated into the primary phloem (pointed by the yellow arrow). Red arrow indicates the protoxylem vessel. Red rectangle, a close look of a developing vascular bundle. (h) Vacuolization of the outer pith cells when the rib meristems were differentiated into the first primary xylem vessels. (i) Pith tissue started to break after the formation of mature vascular bundle (pointed by the red arrow). (l) A developing pith cavity. Ellipse indicates the pith cavity from the axial view. Red arrows indicate the broken trend of pith tissue. Trypan blue (k) and DAB (l) staining of pith tissue in *Bambusa emeiensis* ‘viridiflavus’. 1, vertical sections of *B. emeiensis* ‘viridiflavus’ shoot segments that were treated with ddH_2_O; 2, stained brown sections with DAB; 3, a close look of 2. DAB staining of two other bamboo species, *Ph. incarnata* (m) and *B. multiplex* (n). 1, vertical sections that were treated with ddH_2_O; 2, sections that were treated with DAB. Red arrows indicate the brown pith tissues stained with DAB. (o) Transmission electron microscope observation of pith cells around the pith cavity of *B. emeiensis* ‘viridiflavus’. (1) Chromatin condensing nucleus (pointed by red arrows) in the dying pith cells. (2) Tonoplast ruptures (red arrow), and vesicles were present near the plasma membrane (black arrow), and then a number of vesicles were found in the pith cells (red arrows) (3, 4). (5) Vacuolations (red arrow) emerged in the centre of mitochondrion. (6) Golgi apparatus became unclear and began to degrade. (7) Endoplasmic reticulum became broken (red arrow) and began to degrade, and cytoplasm degraded apparently (star). (8, 9) Apparent degraded materials (red arrows) could be found in the cell lumen. (10) Degraded materials were transported to the nearby cells via plasmodesmata (yellow arrows). (11) Degraded and broken cell walls were apparent in the inner surface of the pith cavity (red arrows). Numbers indicate different cells. (p) Genes related to ROS generation or scavenging, calcium signalling, cell wall modifying and protein degradation were identified from the 150 genes with the highest gene expression levels in the precavity pith cells of *Oligostachyum spongiosum*, a bamboo species with quick separation of pith tissue. CW, cell wall; G, Golgi apparatus; M, mitochondria; N, nucleus; V, vacuole.

To check whether other bamboo species share similar mechanism underlying the pith cavity formation, we investigated the morphologies of pith cavity formation in 33 bamboo species. Interestingly, we found that pith tissues in all investigated bamboo species were collapsed in the top centre parts of the internode before the internode fast growth (Figure 8a,b). Similar to *Ps. japonica*, pith tissue separated slowly in some bamboo species such as *Indocalamus longiauritus* and *Chimonobambusa sichuanensis* (Figure 8a), while in other species such as Moso (*Phyllostachys edulis*) pith tissue separated quickly (Figure 8b).

We then selected a bamboo with quick pith collapse process, Moso bamboo, for cytological analysis. The results showed that Moso pith development followed a regular pattern. Pith cells with the cell division ability appeared apparently before the procambium first appeared (Figure 8c–e). After the rib meristem was first differentiated into the protoxylem, pith cells in the centre started to display a vacuolization phenotype (Figure 8f). The vacuolization continued to strengthen when the rib meristem was first differentiated into the primary phloem (Figure 8g). After the rib meristem was differentiated into the first primary xylem vessels, nearly all outer layers of pith cells were apparently vacuolized (Figure 8h). Pith tissue in the internode centre started to break when the inner vascular bundle was fully formed (Figure 8i). Cells around the pith cavity were broken and displayed a flocculated sludge like shape, and cells near the developing pith cavity were irregular (Figure 8j). Pith tissue was first corrupted at the top‐centre part of internode and then moved towards the bottom part (Figure 8j), which was similar to *Ps. japonica*.

To investigate whether other bamboo species also shared the similar physiological and molecular mechanisms*,* we selected several bamboo species with slow or quick pith collapse for trypan blue staining, H_2_O_2_ accumulation detection and TEM observation as well as transcriptome analysis. Our results showed that both trypan blue and DAB stained pith cells could be found in the precavity pith cells of the three investigated bamboo species including two with quickly collapsed pith tissue (Figure 8k–m) and one with slowly formed pith cavity (Figure 8n). Nuclei with chromatin condensing (Figure 8o1), the ruptured tonoplast (Figure 8o2), an increased number of vesicles aggregated in the cytoplasm (Figure 8o3,4), degraded organelles including mitochondrion (Figure 8o5), golgi apparatus (Figure 8o6) and endoplasmic reticulum (Figure 8o7) as well as degraded cytoplasmic components could also be found in the pith cells around the pith cavity of *Bambusa emeiensis* ‘viridiflavus’ which has quickly collapsed pith tissue. Various degraded materials could be found in the cell lumen during the degradation process (Figure 8o8,9). In some cells degraded materials were found to be transported to nearby cells via plasmodesmata (Figure 8o10). Thin, broken and separated cell walls were also discovered in the inner surface of the pith cavity (Figure 8o11).

To explore the molecular mechanisms underlying the pith cavity formation of different bamboo species, we performed transcriptome sequencing of the pith tissues around pith cavity for *Oligostachyum spongiosum*, a bamboo species with quick separation of pith tissue. Interestingly, among the 150 genes with the highest expression levels, two cell walls modifying, three calcium signalling, ten ROS generation/scavenging and five proteolysis related genes were found in the pith cells of *O. spongiosum* (Table S5; Figure 8p).

### Cellular basis underlying the pith cavity formation variation among different bamboo species

**Figure 9:**
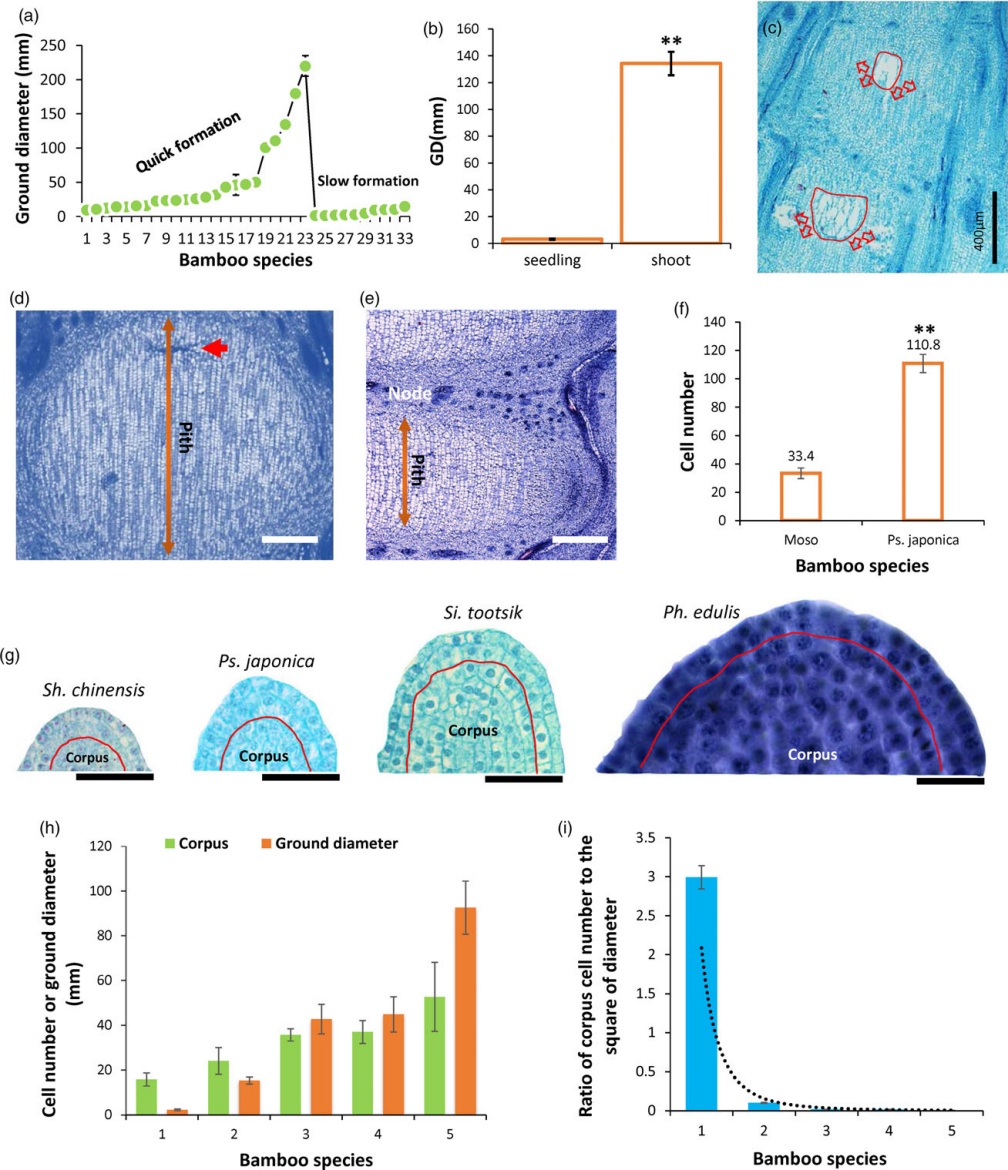
Cellular basis of the slow and quick formation of bamboo pith cavity. (a) Correlation between culm diameter and pith cavity formation rate. Number 1–33 represent *Phyllostachys aureosulcata* ‘Pekinensis’, *Ph. aureosulcata*,* Ph. heteroclada*,* Ph. nigra*,* Ph. bissetii*,* Ph. aurea*,* Ph. arcana* ‘Luteosulcata’, *Semiarundinaria sinica*,* Ph. prominens*,* Ph. glauca*,* Se. densiflora*,* Ph. incarnata*,* Pleioblastus maculatus*,* Ph. glabrata*,* Sinobambusa tootsik*,* Ph. reticulata*,* Ph. iridescens*,* Bambusa emeiensis* ‘viridiflavus’, *Dendrocalamus brandisii*,* De. Hamiltonii*,* Ph. edulis*,* De. Latiflorus*,* De. giganteus*,* Pl. pygmaeus*,* Pl. argenteastriatus*,* Shibataea chinensis* ‘Nakai’, *Pl. fortunei*,* Sasaella kongosanensis* ‘Aureostriatus’, *Indocalamus latifolius*,* Pseudosasa japonica*,* Chimonobambusa sichuanensis*,* In. longiauritus*,* Bambusa multiplex*, respectively. (b) Moso bamboo seedling with small diameter displayed a slowly formed pith cavity (c). Double stars indicate *P *< 0.01. (d) Pith cells across the internodes of *Ps. japonica* and Moso bamboo (e) in which pith cells just started to break (red arrows). Scale bars: 200 μm. (f) Number of pith cells across the internodes of *Ps. japonica* and Moso bamboo. Double stars indicate *P* < 0.01. (g) Shoot apical meristems of four bamboo species with different culm sizes. (h) Cell numbers of corpuses significantly increased with the increase of bamboo ground diameters. Number 1–5 represent *Sh. chinensis*,* Ps. japonica*,* Si. tootsik*,* Ph. nigra* var. *henonis* and *Ph. edulis* (Moso bamboo), respectively. (i) Ratios of corpus cell numbers to the square of bamboo ground diameters decreased exponentially with the increase of diameters. Number 1–5 represent *Sh. chinensis*,* Ps. japonica*,* Si. tootsik*,* Ph. nigra* var. *henonis* and *Ph. edulis*, respectively.

The above results revealed that although different bamboo species shared similar physiological and molecular mechanisms underlying the pith cavity formation process, morphological observation discovered that there were two general types of pith cavity formation among different bamboo species (Figure 8a). Further analysis revealed that the variations in the rates of pith cavity formation were not related to the genus of bamboo, but correlated with culm diameters. Bamboo species with large diameters usually formed pith cavities more rapidly while those with smaller diameters were much slower in pith cavity formation (Figure 9a). Indeed, we found that Moso bamboo young seedlings, which have small culm diameters (Figure 9b), displayed slowly formed pith cavity (Figure 9c), similar to that in *Ps. japonica* (Figure 1b).

To further investigate the underlying cellular basis, we then compared the pith cell numbers across the internode (Figure 9d,e). As expected, pith cell number across the internode of Moso which had a larger culm diameter was dramatically smaller than that of *Ps. japonica* (Figure 9f). We also compared the cell numbers of corpus, which develops to pith tissue and consists of the shoot apical meristem with tunica cells (Reeve, 1948), in five bamboo species with different culm diameters. We found that cell numbers of corpus were significantly larger in species with larger culm diameters (Figure 9g,h), while the ratios of corpus cell numbers to the square of culm diameters reduced exponentially with the increase in culm sizes (Figure 9i).

## Discussion

Our previous work revealed that unexpectedly, pith tissue which will eventually die plays an important role in promoting the primary thickening growth and culm size evolution of bamboo (Wei *et al*., 2017). In this study, using anatomic, mathematic and transcriptomic approaches we extensively explored the morphological, cellular and molecular characteristics during the pith cavity formation, the final developmental stage of pith, to address its formation mechanisms.

### Morphological and cytological characteristics of the pith cavity formation in *Ps. japonica*

Our morphological and cellular observations clearly revealed that the pith cavity of bamboo was formed before the internode fast elongation (Figure 1a), indicating that the cavity formation was not possibly caused by different growth rates of cells. Trypan blue dying and SEM observations of *Ps. japonica* pith tissue also supported this. The formation of pith cavity was resulted from the progressive and regular death of pith cells (Figures 1 and 2), similar to the lysigenous aerenchyma formation of maize roots (Gunawardena *et al*., 2001). The subsequent growth of shoot wall cells in the fast elongation internode thus only expanded the resulting pith cavity. By investigating the cell length along an internode of *Ps. japonica*, we found that shoot wall cells in the top parts of the internode elongated earlier than those of cells in the bottom parts (Figure 1e). These different cell growth behaviours cause the shoot wall cells to produce different extending force to the pith cells, which results in the pith tissues in the top‐centre part of the internode much easier to separate during the internode elongation process (Figure 1i). The spiral growth of bamboo internodes, which is actually the growth of the shoot wall cells, produces anisopleural force on the inside pith tissue, and finally results in the asymmetrical formation of pith cavity (Figure 1d,g,h).

TEM observations further discovered a series of PCD‐related morphological changes as indicated by the rupture of tonoplasts followed by chromatin condensing and degradation, organelle disruption, cytoplasm degradation, plasma‐membrane and cell wall corruptions (Figure 3). DNA cleavage and TUNEL positive nuclei found in the pith cells during pith cavity formation further suggested that PCD might play an important role in this process (Figure 4). The whole process is similar to the programmed cell death during the fistular leaf formation of *Allium fistulosum* (Ni *et al*., 2015).

The above morphological and cytological characteristics also indicate that PCD of pith cells in *Ps. japonica* is a typical ‘vacuolar plant cell death’ according to the classification described in van Doorn *et al*. (2011). However, plasmolysis, a characteristic of necrotic plant cell death (van Doorn *et al*., 2011), was also found in some dying pith cells around the developing pith cavity (Figure 3s,t). We hypothesized that the direct exposure to hollow pith cavity might result in an osmotic pressure to the dying pith cells and finally triggered the plasmolysis, which was also found in cells around the forming aerenchyma in the stems of the waterweed *Egeria densa* (Bartoli *et al*., 2015).

### Molecular characteristics of the pith cavity formation in *Ps. japonica*

So far, most studies of lysigenous aerenchyma formation have been carried out from an anatomical and/or a physiological perspective (Takahashi *et al*., 2015). Molecular mechanisms underlying the lysigenous aerenchyma formation are still poorly understood (Takahashi *et al*., 2015). To further explore the possible molecular basis underlying the cytological changes during the pith cell death of *Ps. japonica*, we analysed transcriptome profiles of pith cells around the pith cavity at different developmental stages. Among the differentially expressed genes between S3/S2 and S1 pith cells, ethylene signalling pathway genes were significantly up‐regulated (Figures 5b,c and 7a). As expected, genes related to Ca^2+^, G‐protein and phospholipase C signalling pathways and ROS burst, which function as the downstream targets of the ethylene pathway (Rajhi *et al*., 2011; Yamauchi *et al*., 2018), were also found to be enhanced in the S3/S2 cells (Figures 5d,e and 7a). H_2_O_2_ was indeed detected in the pith cells during the cavity formation (Figure 7c,d). A number of studies have reported that ethylene, ROS and Ca^2+^ signalling plays important roles in the parenchymal cell death during the aerenchyma formation in rice (Yamauchi *et al*., 2017, 2018), wheat (Yamauchi *et al*., 2014) and maize (Yamauchi *et al*., 2011). For example, Rajhi *et al*. (2011) found that genes associated with Ca^2+^ and reactive oxygen species signalling were induced in the root cortical cells during the lysigenous aerenchyma formation under waterlogged conditions. In rice, death of epidermal cells above nodal adventitious root primordia could be induced by ethylene and H_2_O_2_ (Steffens and Sauter, 2005).

The above PCD‐related upstream signals might trigger a series of cellular processes before cell death via regulating genes involved in various cell metabolisms, which collectively could reduce the synthesis of biomacromolecules such as DNA, protein and lipid, while elevate their degradation (Figures 6a–c and 7a). At the same time, cell wall modifying genes such as pectin esterases and degradation genes such as beta‐1,4‐glucanases (Figures 6d and 7a) were also up‐regulated, which resulted in cell wall loosening and degraded. The degradation products might be transported outside for recycling as transport‐related genes such as amino acid, nitrate and sugar transporters were more active in the S3/S2 pith cells (Figures 6e and 8a). Cell wall modification, proteolysis and transport‐related genes were also found to be induced in cortical cells of maize roots during lysigenous aerenchyma formation under aerobic conditions with ethylene treatment (Takahashi *et al*., 2015).

It is noted that some genes related to the ABA pathway, which can suppress ethylene‐induced plant PCD (Steffens *et al*., 2011), were also up‐regulated in the pith cells at the S3/S2 stages (Figure 5b). These PCD inhibitors might play a brake function in cells around the dying pith cells as not all the pith cells die at the same time.

### Pith cavity formation in different bamboo species

On the basis of the morphological analysis of over 30 bamboo species, we generally divided the pith cavity formation into quick and slow types (Figure 8a). However, according to our investigation, the cellular and physiological as well as molecular mechanisms underlying these two types of pith cavity formation were largely similar. For example, both of them have similar pith collapse patterns with pith cells first collapsed in the top centre part of the internode, and progressively corrupted towards the bottom part of the internode (Figures 1 and 8), and both of them depend on ROS signals (Figure 8k–n). In addition, both of them have similar subcellular changes during pith cell death (Figures 3, 8o). What is more, by investigating the transcriptome profiles of pith tissues around the developing pith cavity in another bamboo species, we found that among the 150 genes with the highest expression level, 20 were related to cell wall modifying, ROS generation/scavenging, calcium signalling and proteolysis, which was similar to the transcriptome files we discovered in *Ps. japonica*.

The slow and quick formation of pith cavity might be resulted from different numbers of pith cell layers across the internode. By investigating the culm diameters among different bamboo species, we found that bamboo species with large culm diameters tended to have the quick collapse of pith tissue (Figure 9a). Bamboo species with small diameters had a greater number of pith cells across the internode than those with large diameters (Figure 9d,e). For example, the number of pith cells across the bottom internode of Moso that has a culm diameter of ~10 cm, in which pith cell began to collapse, was only ~1/3 of that of *Ps. japonica* which has a culm diameter of ~1.0 cm (Figure 9f). Thinner pith cell layers across the internode in the bamboo species with large diameters were much easier to separate than the thicker cell layers in the bamboo species with small diameters when PCD signal was released in the top part of pith tissue (Figures 1b and 8l–n). As we all know, the cross area of bamboo shoot internode increases as the square of the linear dimensions, while the pith cell number increases as the linear dimensions. Thus, with the increase in bamboo shoot diameters, more and more pith cells tended to be assigned to the cross area of bamboo shoot, while less and less pith cells would be distributed to the internode length dimension. This might be why the bamboo species with large diameters usually have less layers of pith cells across the internode than those with small diameters.

By comparing corpus cell numbers among five bamboo species with different culm sizes, we found that although corpus cells, which develops to pith tissue (Wei *et al*., 2017), were dramatically increased with the increase in culm size, and the ratio of corpus cell number to the square of culm diameter was exponentially decreased with the culm size increase (Figure 9g–i), somehow demonstrating that the increase in pith cells could not keep pace with the cross‐area increase. Thus, more pith cells should be reassigned to the cross area to fill the gap, highly possibly by decreasing pith cells across the internode.

In conclusion, our cytological and molecular analyses indicate that the pith cavity of bamboo is formed from the regular death of pith cells, which is a PCD process regulated by ethylene, ROS and calcium signalling and their downstream functional genes such as those involved in DNA degradation, proteolysis, cell wall loosening and degradation as well as nutrition recycling genes such as transport‐related genes. The asymmetrical radial and axial tensile force gradients that were produced by sequential elongation of shoot wall cells from top to bottom part of the internode and the spiral growth of bamboo internode together influenced the pith post‐corruption of bamboo during the formation of pith cavity. The different formation rates of pith cavity in different bamboo species might be caused by different layers of pith cells across the internode which were negatively correlated with the culm diameter.

## Experimental procedures

### Plant materials

*Phyllostachys aureosulcata* ‘Pekinensis’, *Ph. aureosulcata*,* Ph. heteroclada*,* Ph. nigra*,* Ph. bissetii*,* Ph. aurea*,* Ph. arcana* ‘Luteosulcata’, *Semiarundinaria sinica*,* Ph. prominens*,* Ph. glauca*,* Se. densiflora*,* Ph. incarnata*,* Pleioblastus maculatus*,* Ph. glabrata*,* Sinobambusa tootsik*,* Ph. reticulata*,* Ph. iridescens*,* Bambusa emeiensis* ‘viridiflavus’, *Ph. edulis*,* Pl. pygmaeus*,* Pl. argenteastriatus*,* Shibataea chinensis* ‘Nakai’, *Pl. fortunei*,* Sasaella kongosanensis* ‘Aureostriatus’, *Indocalamus latifolius*,* Pseudosasa japonica*,* Chimonobambusa sichuanensis*,* In. longiauritus*,* B. multiplex* and *Oligostachyum spongiosum* were grown in the Bamboo Garden of Nanjing Forestry University, *Dendrocalamus brandisii*,* De. hamiltonii* and *De. giganteus* were collected from Puer City, Yunnan province, and *De. latiflorus* was collected at Xishuangbanna Tropical Botanic Garden in Yunnan province. The annual bamboo shoots were collected for morphological and anatomical analysis. For each bamboo species, at least 30 bamboo plants were selected for ground diameter investigation.

### Light microscopy

Mature Moso bamboo (*Phyllostachys edulis*) shoots immediately emerging from underground and 20‐cm‐tall *Ps. japonica* bamboo shoots were harvested for investigating the cellular process of pith tissue collapse.

Awakening buds (Wei *et al*., 2017) of *Sh. chinensis*,* Ps. japonica*,* Si. tootsik*,* Ph. nigra* var. *henonis* and *Ph. edulis* were used for investigating the SAM morphologies. For SAM morphological data collection, the outer two layers of cells organized regularly were considered as tunica cells, while inner layers of cells usually irregularly organized were regarded as corpus cells in this study. Data were collected from at least three SAM replicates from at least three bamboo buds of each bamboo species, and each SAM replicate was composed of three longitudinal section data.

Methods for making paraffin section and light microscopy observation were same as those described in Wei *et al*. (2017).

### Cell length of the second internode of *Ps. japonica* bamboo shoot

The second internodes with lengths of ~4 cm were sawed into four 1‐cm parts, then fixed in the formalinacetic‐70% alcohol (FAA, v/v) buffer, and exhausted with an aspirator pump. Methods for making paraffin section and light microscopy observation were same as described in Wei *et al*. (2017). The lengths of over 300 parenchymal cells in each 1‐cm part were measured.

### Investigation of the growth pattern of bamboo internode of *Ps. japonica*

The second internode right above the ground was carefully sawed into 10 equal parts. The cross section of each part was scanned from the top by a Scanjet BioScan (RockGene, Shanghai, China). The outlines of the cross sections were extracted using the magnetic lasso tool in Photoshop (Adobe, San Jose, CA, USA). The MATLAB and R functions were used to describe the outside contours of internode cross‐sections as described in Wei *et al*. (2018) and Shi *et al*. (2015).

### Scanning electron microscopy observation of *Ps. japonica* pith tissues

Pith tissues of *Ps. japonica* at different stages were first fixed in the FAA buffer, and then modified by sharp double‐edge razor blade into thin and smooth sections, which were subsequently dehydrated using graded ethanol. After drying, conventional sections were made and observed under a JEOL JSM‐6300 scanning electron microscopy (SEM) (JEOL, Tokyo, Japan).

### Transmission electron microscopy of *Ps. japonica* pith tissues

Pith tissues around pith cavity of *Ps. japonica* at three developmental stages (Figure 4a) were collected and fixed in 2% paraformaldehyde and 1% glutaraldehyde for 4 h at 20 °C. Methods for TEM section making and observation were same as described those in Wei *et al*. (2017).

### DNA cleavage, DAPI staining and TUNEL analysis of *Ps. japonica* pith tissues

DNA of *Ps. japonica* pith tissues at different stages were extracted using the HF224‐01 Kit (Yuanpinghao Biotech, China). Electrophoresis was then carried out to check the cleavage of the DNA.

For DAPI and TUNEL assays, pith tissue samples were first fixed in formalinacetic‐70% alcohol (FAA, v/v) buffer and exhausted with an aspirator pump. Serial transverse sections (7 μm thick) from paraffin embedded tissues were made. The resulting sections were then dewaxed for TUNEL assay and DAPI staining. The TUNEL and DAPI assays were carried out according to the instructions of *In situ* Apoptosis Detection Kit (KGA7073) (Keygen Biotech, Nanjing, China). Subsequently, these sections were observed under a fluorescence microscope Leica DM2500 light microscope (Leica, Wetzlar, Germany).

### RNA extraction and transcriptome sequencing of *Ps. japonica* pith tissue

Pith tissues around pith cavity of *Ps. japonica* at three developmental stages (Figure 4a), each stage with six biological replicates, were carefully excised, and then ground into powder in liquid nitrogen. Total RNA was extracted using the RNAprep Pure Kit (DP441) (TIANGEN Biotechnology, Beijing, China). The ratio of OD260 and OD280 of the extracted RNA was determined with a NanoDrop 1000 spectrophotometer (Thermo Scientific, Waltham, MA, USA). Electrophoresis was also performed to examine the RNA quality. RNA samples were further checked for the RNA Integrity Number (RIN) values on an Agilent Bioanalyzer 2100 system (Agilent Technologies, Palo Alto, CA, USA).

Strand‐specific RNA‐Seq library preparation and sequencing were both performed at Novogene Biotech (Beijing, China) using standard Illumina protocols. RNA‐Seq libraries were sequenced on an Illumina HiSeq 2500 system with the paired‐end mode. Raw sequence reads have been deposited into the NCBI sequence read archive (SRA) under accession SRP144234.

### Transcriptome sequence processing, assembly and annotation

Raw RNA‐Seq reads were first processed to remove adaptors and low‐quality sequences using Trimmomatic (Bolger *et al*., 2014). The resulting reads shorter than 40 bp were discarded. The remaining reads were aligned to the ribosomal RNA (rRNA) database (Quast *et al*., 2013) using Bowtie (Langmead *et al*., 2009), and reads that could be aligned were discarded. The final cleaned reads of all samples were combined, and *de novo* assembled into contigs using rnaSPAdes with default parameters (Bankevich *et al*., 2012). CD‐HIT v 4.7 was used to remove redundancy of the rnaSPAdes‐assembled contigs, with sequence identity cut‐off set to 97% (Fu *et al*., 2012). BUSCO alignment was carried out to evaluate the quality of the assembled transcripts (Simao *et al*., 2015; Waterhouse *et al*., 2017). The transcripts were finally annotated by comparing their sequences against the Swiss‐Prot and TrEMBL databases using BLASTx with an e‐value cut‐off of 1e^−5^.

### Transcript abundance analysis of *Ps. japonica* pith tissue

The cleaned reads were aligned to the transcript assembly using the Bowtie2 program (v2.3.4.1) (Langmead and Salzberg, 2012). Following alignments, raw read counts for each transcript were derived by using RSEM (v1.2.31) (Li and Dewey, 2011) and then normalized to FPKM (fragments per kilobase exon per million mapped fragments). Raw counts were fed to the DESeq2 package (Love *et al*., 2014) to identify DEGs, which were defined as those with adjusted *P* < 0.05 and fold changes no < 2. MapMan (v.3.5.1R2) was used for visualizing expression patterns of DEGs (Thimm *et al*., 2004). The mapping file of assembled transcripts was generated using Mercator (Lohse *et al*., 2014).

### Quantitative real‐time PCR

For qPCR, same total RNA from the pith tissues of *Ps. japonica* used for transcriptome sequencing was used. One microgram of total RNA was transcribed in a total volume of 20 μL solution as described in the operation manual of the PrimeScript^TM^ RT reagent Kit (Takara, Code No. RR047A). qPCR was performed using the TransStart Tip Green qPCR SuperMix Kit (Transgene Biotech, Beijing China) on an ABI StepOne Plus Real‐Time PCR System (Applied Biosystems) according to the manufacturers’ instructions. The experiments were repeated technically at least three times. The relative abundance of each gene was calculated from the 2^−▵Cq^ values between the target gene and the reference gene (three replicates for each gene) (Livak and Schmittgen, 2001). Transcription initiation factor IIE (TFIIE) gene was used as the internal control (Yamauchi *et al*., 2017). Gene‐specific primers used for qPCR are provided in Table S6.

### Hydrogen peroxide detection in the pith tissues of *Ps. japonica*

Detection of H_2_O_2_ using the 3,3′‐diaminobenzidine (DAB) liquid substrate system was performed according to the manufacturer's instructions (Sangon Biotech, Shanghai, China). Internodal vertical‐sections including pith cavities at different developmental stages were stained with the DAB solution for 5 min, and then observed under a fluorescence microscope Leica DM2500 light microscope (Leica, Wetzlar, Germany).

### Morphological, physiological and transcriptome analysis of pith cavity formation in different bamboo species

Thirty‐three bamboo species described above were used for investigating morphological variations of pith cavity formation. Four bamboo species were selected for investigating physiological changes or transcriptome profiles underlying their pith cavity formation, including *Bambusa emeiensis* ‘viridiflavus’ for trypan blue staining, H_2_O_2_ detection and transmission electron microscopy observation, and *Phyllostachys incarnata* and *Bambusa multiplex* for H_2_O_2_ detection. Methods for trypan blue staining, H_2_O_2_ detection and transmission electron microscopy observation were same as described above.

For transcriptome analysis, pith tissues around pith cavity of *Oligostachyum spongiosum,* a bamboo species with quick formation of pith cavity, were collected. Transcriptome data processing and analysis were same as described above.

## Conflict of interest

The authors declare no financial or commercial conflict of interest.

## Supporting information


**Table S1** Summary of Illumina paired‐end reads of *Ps. japonica*.

**Table S2** Summary of BUSCO alignment results of *Ps. japonica*.

**Table S3** Correlation of gene expression profiles between biological replicates of *Ps. japonica*.

**Table S4** Differentially expressed genes in the pith cells of *Ps. japonica* between different stages.

**Table S5** Top 150 genes with the highest expression levels in precavity pith cells of *Oligostachyum spongiosum*.

**Table S6** Primers used for qPCR analysis.

## References

[pbi13033-bib-0001] Bankevich, A. , Nurk, S. , Antipov, D. , Gurevich, A.A. , Dvorkin, M. , Kulikov, A.S. , Lesin, V.M. *et al*. (2012) SPAdes: a new genome assembly algorithm and its applications to single‐cell sequencing. J. Comput. Biol. 19, 455–477. 22506599 10.1089/cmb.2012.0021 PMC3342519

[pbi13033-bib-0002] Bartoli, G. , Forino, L.M. , Durante, M. and Tagliasacchi, A.M. (2015) A lysigenic programmed cell death‐dependent process shapes schizogenously formed aerenchyma in the stems of the waterweed Egeria densa. Ann. Bot. 116, 91–99. 26002256 10.1093/aob/mcv067 PMC4479754

[pbi13033-bib-0003] Bolger, A.M. , Lohse, M. and Usadel, B. (2014) Trimmomatic: a flexible trimmer for illumina sequence data. Bioinformatics, 30, 2114–2120. 24695404 10.1093/bioinformatics/btu170 PMC4103590

[pbi13033-bib-0004] van Doorn, W.G. , Beers, E.P. , Dangl, J.L. , Franklin‐Tong, V.E. , Gallois, P. , Hara‐Nishimura, I. , Jones, A.M. *et al*. (2011) Morphological classification of plant cell deaths. Cell Death Differ. 18, 1241–1246. 21494263 10.1038/cdd.2011.36 PMC3172093

[pbi13033-bib-0005] Fu, L. , Niu, B. , Zhu, Z. , Wu, S. and Li, W. (2012) CD‐HIT: accelerated for clustering the next‐generation sequencing data. Bioinformatics, 28, 3150–3152. 23060610 10.1093/bioinformatics/bts565 PMC3516142

[pbi13033-bib-0006] Gao, J. , Zhang, Y. , Zhang, C.L. , Qi, F.Y. , Li, X.P. , Mu, S.H. and Peng, Z.H. (2014) Characterization of the floral transcriptome of moso bamboo (*Phyllostachys edulis*) at different flowering developmental stages by transcriptome sequencing and RNA‐seq analysis. PLoS ONE, 9, e98910. 24915141 10.1371/journal.pone.0098910 PMC4051636

[pbi13033-bib-0500] Gielis, J. (2003) A generic geometric transformation that unifies a wide range of natural and abstract shapes. Am. J. Bot., 90, 333‐338. 21659124 10.3732/ajb.90.3.333

[pbi13033-bib-0007] Gunawardena, A.H. , Pearce, D.M. , Jackson, M.B. , Hawes, C.R. and Evans, D.E. (2001) Characterisation of programmed cell death during aerenchyma formation induced by ethylene or hypoxia in roots of maize (*Zea mays* L.). Planta, 212, 205–214. 11216841 10.1007/s004250000381

[pbi13033-bib-0008] He, C.Y. , Cui, K. , Zhang, J.G. , Duan, A.G. and Zeng, Y.F. (2013) Next‐generation sequencing‐based mRNA and microRNA expression profiling analysis revealed pathways involved in the rapid growth of developing culms in moso bamboo. BMC Plant Biol. 13, 119. 23964682 10.1186/1471-2229-13-119 PMC3765735

[pbi13033-bib-0009] Langmead, B. and Salzberg, S.L. (2012) Fast gapped‐read alignment with Bowtie 2. Nat. Methods, 9, 357–359. 22388286 10.1038/nmeth.1923 PMC3322381

[pbi13033-bib-0501] Langmead, B. , Trapnell, C. , Pop, M. and Salzberg, S.L. (2009) Ultrafast and memory‐efficient alignment of short DNA sequences to the human genome. Genome Biol, 10, R25. 10.1186/gb-2009-10-3-r25 19261174 PMC2690996

[pbi13033-bib-0010] Li, B. and Dewey, C.N. (2011) RSEM: accurate transcript quantification from RNA‐Seq data with or without a reference genome. BMC Bioinformatics, 12, 16. 21816040 10.1186/1471-2105-12-323 PMC3163565

[pbi13033-bib-0011] Li, L. , Cheng, Z. , Ma, Y. , Bai, Q. , Li, X. , Cao, Z. , Wu, Z. *et al*. (2018) The association of hormone signalling genes, transcription and changes in shoot anatomy during moso bamboo growth. Plant Biotechnol. J. 16, 72–85. 28499069 10.1111/pbi.12750 PMC5785349

[pbi13033-bib-0502] Liang, F. , Shen, L.Z. , Chen, M. and Yang, Q. (2008) Formation of intercellular gas space in the diaphragm during the development of aerenchyma in the leaf petiole of *Sagittaria trifolia* . Aquat. Bot., 88, 185–195.

[pbi13033-bib-0012] Liu, M.Y. , Qiao, G.R. , Jiang, J. , Yang, H.Q. , Xie, L.H. , Xie, J.Z. and Zhuo, R.Y. (2012) Transcriptome sequencing and *de novo* analysis for ma bamboo (*Dendrocalamus latiflorus* Munro) using the illumina platform. PLoS ONE, 7, e46766. 23056442 10.1371/journal.pone.0046766 PMC3463524

[pbi13033-bib-0013] Livak, K.J. and Schmittgen, T.D. (2001) Analysis of relative gene expression data using real‐time quantitative PCR and the 2(T)(‐Delta Delta C) method. Methods, 25, 402–408. 11846609 10.1006/meth.2001.1262

[pbi13033-bib-0014] Lohse, M. , Nagel, A. , Herter, T. , May, P. , Schroda, M. , Zrenner, R. , Tohge, T. *et al*. (2014) Mercator: a fast and simple web server for genome scale functional annotation of plant sequence data. Plant Cell Environ. 37, 1250–1258. 24237261 10.1111/pce.12231

[pbi13033-bib-0015] Love, M. , Anders, S. and Huber, W. (2014) Differential analysis of count data–the DESeq2 package. Genome Biol. 15, 550. 25516281 10.1186/s13059-014-0550-8 PMC4302049

[pbi13033-bib-0016] Ni, X.L. , Meng, Y. , Zheng, S.S. and Liu, W.Z. (2014) Programmed cell death during aerenchyma formation in *Typha angustifolia* leaves. Aquat. Bot. 113, 8–18.

[pbi13033-bib-0017] Ni, X.L. , Su, H. , Zhou, Y.F. , Wang, F.H. and Liu, W.Z. (2015) Leaf‐shape remodeling: programmed cell death in fistular leaves of *Allium fistulosum* . Physiol. Plant. 153, 419–431. 25132341 10.1111/ppl.12255

[pbi13033-bib-0018] Peng, Z. , Zhang, C. , Zhang, Y. , Hu, T. , Mu, S. , Li, X. and Gao, J. (2013) Transcriptome sequencing and analysis of the fast growing shoots of moso bamboo (*Phyllostachys edulis*). PLoS ONE, 8, e78944. 24244391 10.1371/journal.pone.0078944 PMC3820679

[pbi13033-bib-0019] Qiao, G.R. , Yang, H.Q. , Zhang, L. , Han, X.J. , Liu, M.Y. , Jiang, J. , Jiang, Y.C. *et al*. (2014) Enhanced cold stress tolerance of transgenic *Dendrocalamus latiflorus* Munro (Ma bamboo) plants expressing a bacterial CodA gene. In Vitro Cell Dev. Biol. Plant, 50, 385–391.

[pbi13033-bib-0020] Quast, C. , Pruesse, E. , Yilmaz, P. , Gerken, J. , Schweer, T. , Yarza, P. , Peplies, J. *et al*. (2013) The SILVA ribosomal RNA gene database project: improved data processing and web‐based tools. Nucleic Acids Res. 41, D590–D596. 23193283 10.1093/nar/gks1219 PMC3531112

[pbi13033-bib-0021] Rajhi, I. , Yamauchi, T. , Takahashi, H. , Nishiuchi, S. , Shiono, K. , Watanabe, R. , Mliki, A. *et al*. (2011) Identification of genes expressed in maize root cortical cells during lysigenous aerenchyma formation using laser microdissection and microarray analyses. New Phytol. 190, 351–368. 21091694 10.1111/j.1469-8137.2010.03535.x

[pbi13033-bib-0022] Reeve, R.M. (1948) The ‘Tunica‐Corpus’ concept and development of shoot apices in certain dicotyledons. Am. J. Bot. 35, 65–75.

[pbi13033-bib-0503] Shi, P.J. , Huang, J.G. , Hui, C. , Grissino‐Mayer, H.D. , Tardif, J.C. , Zhai, L.H. , Wang, F.S. , *et al*. (2015) Capturing spiral radial growth of conifers using the superellipse to model tree‐ring geometric shape. Front Plant Sci., 6, 856. 10.3389/fpls.2015.00856 26528316 PMC4606055

[pbi13033-bib-0023] Shih, M.C. , Chou, M.L. , Yue, J.J. , Hsu, C.T. , Chang, W.J. , Ko, S.S. , Liao, D.C. *et al*. (2014) BeMADS1 is a key to delivery MADSs into nucleus in reproductive tissues‐*de novo* characterization of *Bambusa edulis* transcriptome and study of MADS genes in bamboo floral development. BMC Plant Biol. 14, 179. 24989161 10.1186/1471-2229-14-179 PMC4087239

[pbi13033-bib-0024] Simao, F.A. , Waterhouse, R.M. , Ioannidis, P. , Kriventseva, E.V. and Zdobnov, E.M. (2015) BUSCO: assessing genome assembly and annotation completeness with single‐copy orthologs. Bioinformatics, 31, 3210–3212. 26059717 10.1093/bioinformatics/btv351

[pbi13033-bib-0025] Steffens, B. and Sauter, M. (2005) Epidermal cell death in rice is regulated by ethylene, gibberellin, and abscisic acid. Plant Physiol. 139, 713–721. 16169967 10.1104/pp.105.064469 PMC1255990

[pbi13033-bib-0026] Steffens, B. , Geske, T. and Sauter, M. (2011) Aerenchyma formation in the rice stem and its promotion by H_2_O_2_ . New Phytol. 190, 369–378. 21039565 10.1111/j.1469-8137.2010.03496.x

[pbi13033-bib-0027] Takahashi, H. , Yamauchi, T. , Rajhi, I. , Nishizawa, N.K. and Nakazono, M. (2015) Transcript profiles in cortical cells of maize primary root during ethylene‐induced lysigenous aerenchyma formation under aerobic conditions. Ann. Bot. 115, 879–894. 25858325 10.1093/aob/mcv018 PMC4407059

[pbi13033-bib-0028] Thimm, O. , Blasing, O. , Gibon, Y. , Nagel, A. , Meyer, S. , Kruger, P. , Selbig, J. *et al*. (2004) MAPMAN: a user‐driven tool to display genomics data sets onto diagrams of metabolic pathways and other biological processes. Plant J. 37, 914–939. 14996223 10.1111/j.1365-313x.2004.02016.x

[pbi13033-bib-0029] Thompson, D.A.W. (1945) On Growth and Form. New York: University Press, MacMillan in Cambridge.

[pbi13033-bib-0030] Waterhouse, R.M. , Seppey, M. , Simao, F.A. , Manni, M. , Ioannidis, P. , Klioutchnikov, G. , Kriventseva, E.V. *et al*. (2017) BUSCO applications from quality assessments to gene prediction and phylogenomics. Mol. Biol. Evol. 35, 543–548. 10.1093/molbev/msx319 PMC5850278 29220515

[pbi13033-bib-0031] Wei, Q. , Cao, H.M. , Li, Z.R. , Kuai, B.K. and Ding, Y.L. (2013) Identification of an AtCRN1‐like chloroplast protein BeCRN1 and its distinctive role in chlorophyll breakdown during leaf senescence in bamboo (*Bambusa emeiensis* ‘Viridiflavus’). Plant Cell Tissue Organ Cult. 114, 1–10.

[pbi13033-bib-0032] Wei, Q. , Cao, J.J. , Qian, W.J. , Xu, M.J. , Li, Z.R. and Ding, Y.L. (2015) Establishment of an efficient micropropagation and callus regeneration system from the axillary buds of *Bambusa ventricosa* . Plant Cell Tissue Organ Cult. 122, 1–8.

[pbi13033-bib-0033] Wei, Q. , Jiao, C. , Guo, L. , Ding, Y. , Cao, J. , Feng, J. , Dong, X. *et al*. (2017) Exploring key cellular processes and candidate genes regulating the primary thickening growth of Moso underground shoots. New Phytol. 214, 81–96. 27859288 10.1111/nph.14284

[pbi13033-bib-0034] Wei, Q. , Jiao, C. , Ding, Y. , Gao, S. , Guo, L. , Chen, M. , Hu, P. *et al*. (2018) Cellular and molecular characterizations of a slow‐growth variant provide insights into the fast growth of bamboo. Tree Physiol. 38, 641–654. 29077967 10.1093/treephys/tpx129

[pbi13033-bib-0035] Yamauchi, T. , Rajhi, I. and Nakazono, M. (2011) Lysigenous aerenchyma formation in maize root is confined to cortical cells by regulation of genes related to generation and scavenging of reactive oxygen species. Plant Signal. Behav. 6, 759–761. 21502817 10.4161/psb.6.5.15417 PMC3172858

[pbi13033-bib-0036] Yamauchi, T. , Watanabe, K. , Fukazawa, A. , Mori, H. , Abe, F. , Kawaguchi, K. , Oyanagi, A. *et al*. (2014) Ethylene and reactive oxygen species are involved in root aerenchyma formation and adaptation of wheat seedlings to oxygen‐deficient conditions. J. Exp. Bot. 65, 261–273. 24253196 10.1093/jxb/ert371 PMC3883296

[pbi13033-bib-0037] Yamauchi, T. , Yoshioka, M. , Fukazawa, A. , Mori, H. , Nishizawa, N.K. , Tsutsumi, N. , Yoshioka, H. *et al*. (2017) An NADPH oxidase RBOH functions in rice roots during lysigenous aerenchyma formation under oxygen‐deficient conditions. Plant Cell, 29, 775–790. 28351990 10.1105/tpc.16.00976 PMC5435434

[pbi13033-bib-0038] Yamauchi, T. , Colmer, T.D. , Pedersen, O. and Nakazono, M. (2018) Regulation of root traits for internal aeration and tolerance to soil waterlogging‐flooding stress. Plant Physiol. 176, 1118–1130. 29118247 10.1104/pp.17.01157 PMC5812745

[pbi13033-bib-0039] Ye, S. , Cai, C. , Ren, H. , Wang, W. , Xiang, M. , Tang, X. , Zhu, C. *et al*. (2017) An efficient plant regeneration and transformation system of ma bamboo (*Dendrocalamus latiflorus* Munro) started from young shoot as explant. Front Plant Sci. 8, 1298. 28798758 10.3389/fpls.2017.01298 PMC5529393

[pbi13033-bib-0040] Zhang, X.M. , Zhao, L. , Larson‐Rabin, Z. , Li, D.Z. and Guo, Z.H. (2012) *De novo* sequencing and characterization of the floral transcriptome of *Dendrocalamus latiflorus* (Poaceae: Bambusoideae). PLoS ONE, 7, e42082. 22916120 10.1371/journal.pone.0042082 PMC3419236

