# Supplementary material for: Morphological dissection and cellular and transcriptome characterizations of bamboo pith cavity formation reveal a pivotal role of genes related to programmed cell death
Source: Plant Biotechnol J. 2018 Dec 9;17(5):982–97. doi: 10.1111/pbi.13033 (PMC6587456; doi:10.1111/pbi.13033)
Supplement: Supplementary file 1 — Table S1 Summary of Illumina paired‐end reads of Ps. japonica. [file PBI-17-982-s006.docx]

**Table S1 Summary of Illumina paired-end reads of *Ps. japonica.***

| **Sample ID** | **No. Raw read pairs** | **No. Raw read pairs** | **No. mapped read pairs** | **NCBI accession number** |
| --- | --- | --- | --- | --- |
| S1-1 | 19,694,986 | 18,585,040 | 16,234,171 | SRR7090629 |
| S1-2 | 14,200,037 | 12,953,499 | 11,194,248 | SRR7090630 |
| S1-3 | 19,437,325 | 18,293,888 | 15,742,148 | SRR7090631 |
| S1-4 | 15,624,645 | 14,269,808 | 12,349,930 | SRR7090632 |
| S1-5 | 15,324,639 | 13,705,723 | 11,801,080 | SRR7090633 |
| S1-6 | 16,010,114 | 14,172,970 | 12,256,587 | SRR7090634 |
| S2-1 | 15,271,637 | 13,572,067 | 11,711,792 | SRR7090635 |
| S2-2 | 15,618,099 | 14,164,446 | 12,309,454 | SRR7090636 |
| S2-3 | 16,547,059 | 15,157,100 | 13,187,227 | SRR7090637 |
| S2-4 | 18,252,866 | 16,361,195 | 14,219,410 | SRR7090638 |
| S2-5 | 14,834,399 | 13,322,989 | 11,544,096 | SRR7090639 |
| S2-6 | 18,458,609 | 16,467,447 | 14,278,659 | SRR7090640 |
| S3-1 | 16,687,702 | 14,674,852 | 12,844,276 | SRR7090641 |
| S3-2 | 19,530,511 | 17,525,380 | 15,259,811 | SRR7090642 |
| S3-3 | 16,666,920 | 14,659,856 | 12,343,822 | SRR7090643 |
| S3-4 | 16,271,346 | 14,775,711 | 12,813,441 | SRR7090644 |
| S3-5 | 17,649,216 | 15,860,977 | 13,689,155 | SRR7090645 |
| S3-6 | 17,596,214 | 16,048,697 | 14,040,462 | SRR7090646 |
| **Total** | **303,676,324** | **274,571,645** | **237,819,769** |  |

**Table S6 Primers used for qPCR analysis of *Psudosasa japonica* genes**

| **Gene name** | **Forward primer** (5'->3') | **Reverse primer** (5'->3') |
| --- | --- | --- |
| *AP2/EREBP* | ATTTGAGCCTGGGGGTGC | GTGTCGCTCTGCTCTTCCTCA |
| *RBOH* | ACCTCCGACATTCCGTTACAAG | CTGCTGAGAATACCCGCTTGA |
| *CaLB* | GCATTCGGGCAGACATTAGTT | GGACCAAGACGACCTTTCACA |
| *CaBP* | CGGAGTTCGTGAGGTTGGTG | ATCGTTGATGTCCTGCTCCG |
| *XCP2* | CTTCTCCATTGTGGGCTACTCC | TGTTGATCTCGTCGATGTGCTT |
| *UBL5* | GTCTGGGGAAGAAGGTGCG | TCTCATAGTCGGCGAGGGTG |
| *EXPB3* | GTCTACTTCGCGGTGCTCG | TTGCCGGACTCGTTGGTG |
| *PMI* | CTGTCGTTGGGCAAGGGTT | GAGGGAGTGGGCGTAGAGTGT |
| *AAP2* | GGCGAAGGTGATGAGGAAGG | CGACGAGGTGGACGACGAT |
| *TIP4-1* | GGCACATCTCGGCGTTCA | AGACCACGAAGAGGAGGGAGA |
| *TFIIE-1* | GCAGGTGAAGTTTGGCTGTTATC | CGCCGCTTGGTAGTATTGGT |

*AP2/EREBP*: ethylene response factor; *RBOH*: respiratory burst oxidase protein; *CaBP*: calcium-binding EF-hand family protein; *CaLB*: calcium-dependent lipid-binding protein; *XCP2*: xylem cysteine peptidase 2; *UBL5*: ubiquitin-like protein 5; *EXPB3*: beta expansin; *PMI*: pectin methylesterase inhibitor; *AAP2*: amino acid permease 2; *TIP4-1*: tonoplast intrinsic protein 4-1; *TFIIE*: transcription initiation factor TFIIE
