# Supplementary material for: Morphological dissection and cellular and transcriptome characterizations of bamboo pith cavity formation reveal a pivotal role of genes related to programmed cell death
Source: Plant Biotechnol J. 2018 Dec 9;17(5):982–97. doi: 10.1111/pbi.13033 (PMC6587456; doi:10.1111/pbi.13033)
Supplement: Supplementary file 6 — Table S6 Primers used for qPCR analysis. [file PBI-17-982-s003.docx]

**Table S6 Primers used for qPCR analysis**

| **Gene name** | **Forward primer** (5'->3') | **Reverse primer** (5'->3') |
| --- | --- | --- |
| *AP2/EREBP* | ATTTGAGCCTGGGGGTGC | GTGTCGCTCTGCTCTTCCTCA |
| *RBOH* | ACCTCCGACATTCCGTTACAAG | CTGCTGAGAATACCCGCTTGA |
| *CaLB* | GCATTCGGGCAGACATTAGTT | GGACCAAGACGACCTTTCACA |
| *CaBP* | CGGAGTTCGTGAGGTTGGTG | ATCGTTGATGTCCTGCTCCG |
| *XCP2* | CTTCTCCATTGTGGGCTACTCC | TGTTGATCTCGTCGATGTGCTT |
| *UBL5* | GTCTGGGGAAGAAGGTGCG | TCTCATAGTCGGCGAGGGTG |
| *EXPB3* | GTCTACTTCGCGGTGCTCG | TTGCCGGACTCGTTGGTG |
| *PMI* | CTGTCGTTGGGCAAGGGTT | GAGGGAGTGGGCGTAGAGTGT |
| *AAP2* | GGCGAAGGTGATGAGGAAGG | CGACGAGGTGGACGACGAT |
| *TIP4-1* | GGCACATCTCGGCGTTCA | AGACCACGAAGAGGAGGGAGA |
| *TFIIE-1* | GCAGGTGAAGTTTGGCTGTTATC | CGCCGCTTGGTAGTATTGGT |

*AP2/EREBP*: ethylene response factor; *RBOH*: respiratory burst oxidase protein; *CaBP*: calcium-binding EF-hand family protein; *CaLB*: calcium-dependent lipid-binding protein; *XCP2*: xylem cysteine peptidase 2; *UBL5*: ubiquitin-like protein 5; *EXPB3*: beta expansin; *PMI*: pectin methylesterase inhibitor; *AAP2*: amino acid permease 2; *TIP4-1*: tonoplast intrinsic protein 4-1; *TFIIE*: transcription initiation factor TFIIE
